# microRNA and Other Small RNA Sequence Profiling across Six Tissues of Chinese Forest Musk Deer (*Moschus berezovskii*)

**DOI:** 10.1155/2019/4370704

**Published:** 2019-05-12

**Authors:** Hang Jie, Pu Zhang, Zhongxian Xu, Shailendra Kumar Mishra, Meiyan Lei, Dejun Zeng, Guijun Zhao, Diyan Li

**Affiliations:** ^1^Provincial Key Laboratory for Farm Animal Genetic Resource Exploration and Innovation, Sichuan Agricultural University, Chengdu, China; ^2^Chongqing Engineering Technology Research Center for GAP of Genuine Medicinal Materials, Chongqing Institute of Medicinal Plant Cultivation, Nanchuan, Chongqing, China

## Abstract

The Chinese forest musk deer (*Moschus berezovskii*) is an economically important species distributed throughout southwest China and northern Vietnam. Occurrence and development of disease are aggravated by inbreeding and genetic diversity declines in captive musk deer populations. Deep transcriptomics investigation may provide a promising way to improve genetic health of captive and wild FMD population. MicroRNAs (miRNAs), which regulate gene expression by targeting and suppressing of mRNAs, play an important role in physiology and organism development control. In this study, RNA-seq technology was adopted to characterize the miRNA transcriptome signature among six tissues (heart, liver, spleen, lung, kidney, and muscle) in Chinese forest musk deer at two years of age. Deep sequencing generated a total of 103,261,451 (~87.87%) good quality small RNA reads; of them 6,622,520 were unique across all six tissues. A total of 2890 miRNAs were identified, among them 1129 were found to be expressed in all tissues. Moreover, coexpression of 20 miRNAs (>2000RPM) in all six tissues and top five highly expressed miRNAs in each tissue implied the crucial and particular function of them in FMD physiological processes. Our findings of forest musk deer miRNAs supplement the database of transcriptome information for this species and conduce to our understanding of forest musk deer biology.

## 1. Introduction

The forest musk deer (*Moschus berezovskii*, FMD) is commercially famous for musk production, due to its cosmetic (perfume industry) and alleged pharmaceutical properties [1]. Notably, it is regrettable that the musk was secreted by preputial gland of adult male FMD in a very limited amount. In past decades, the population of wild FMD has been declined rapidly due to considerable poaching and massive habitat destruction and become extraordinarily endangered. The wild FMD listed in Appendix II in CITES and under class I state key protection in China (http://www.iucnredlist.org/). Since the 1960s, FMD farms have been set up to conserve wild populations and obtain musk resources in a safe and sustainable way in China [2]. However, survival of captive musk deer is considerably affected by high incidence of diseases, like Diarrhea, gastrointestinal diseases, dyspepsia, pneumonia, metritis, urinary stones, and abscesses which gravely affect the population growth [3].

MiRNAs, belonging to small, noncoding RNAs (short sequence of 18-25nt), have a vital role in posttranscriptional mechanism by recognizing specific mRNA targets [4]. Thus, microRNAs expression profiling is of global interest due to their key role in the regulation of miscellaneous biological processes, such as body development, cell differentiation, proliferation, apoptosis, immune response, reproductive system development, gametogenesis, and organogenesis [5–9], although few transcriptomics had been made to demonstrate the expression of miRNA in heart, musk gland, and blood of FMD [10, 11]. Thus, it is essential to establish depth exploration of miRNA transcriptome across a wide range tissue in FMD. To date, a total of 38,589 mature miRNAs sequence have been discovered from 271 species (miRBase 22, July 2018). Recently, the first genome sequence and gene annotation for the FMD has been published [12]. The study of the FMD miRNAs and their interactions with target genes will provide further insight into several physiological processes of FMD.

In this study, we carried out the high-throughput sequencing analysis on small RNAs of six tissues (heart, liver, spleen, lung, kidney, and muscle) of forest musk deer (*Moschus berezovskii*) using the RNA-seq technique on the Hi-seq 2500 sequencing platform. The aim of this study was to discover and characterize FMD specific miRNA in a large number of tissues in order to provide an extensive repertoire of expression, illustrating the potential role of miRNAs and their targets on FMD biological processes.

## 2. Materials and Methods

### 2.1. Sample Collection and RNA Extraction

The experimental Chinese forest musk deer was reared in Chongqing Institute of Medicinal Plant Cultivation (Chongqing, China). The six tissues (heart, liver, spleen, lung, kidney and muscle) were collected from one sexually mature male individual, which died from earthquake at April 20^th^, 2013. Total RNA was extracted from six samples with RNAiso reagent (TaKaRa, Japan) according to the manufacturer's instructions. Following isolation, the purity (absorbance ratios, 260/280) and yield of RNA were determined using with a Qubit 3.0 Fluorometer (Thermo Fisher Scientific, Waltham, USA) and the RNA integrity number (RIN) [13] was determined using the Agilent Bioanalyzer, which reported an RNA integrity number of >8.5 for all samples. Total RNA samples were stored at −80°C until use. This study was carried out in accordance with the recommendations of the Animal Care and Committee of Sichuan Agricultural University under permit number DKY-20145020.

### 2.2. Small RNA Sequencing and Data Analysis

The purified RNA samples were sent for sequencing (RNA-seq) on Hi-seq 2500 sequencing platform at Sangon Biotech (Shanghai) Co. Ltd. (Shanghai), China. After getting the raw data, Cutadapt v1.9.1 software [14] used to remove the ligated adapter sequences. Additional filtering was applied to discard low-quality reads, insufficient and overlong sequences. rRNAs, tRNAs, snoRNAs, snRNAs, and other noncoding RNAs were distinguished and eliminated based on reference gene annotated in GenBank (http://www.ncbi.nlm.nih.gov/) and Rfam databases (http://rfam.janelia.org/). Known miRNAs in all samples were identified by comparison with the specified range in the miRBase version 22 (http://www.mirbase.org). Through BLASTN, the remaining reads were compared with all nonredundant mature miRNAs, which are obtained from miRBase 20.0 database (http://www.mirbase.org/) [15]. Fragment per kilobase of exon per million fragments mapped (FPKM) values was used to evaluate differential expression [16].* P* values were calculated using multiple hypothesis testing.* P*⩽ 0.05 and |Log2FoldChange| *⩾*1 were employed to evaluate differentially expressed miRNAs. Next, miRanda (http://www.microrna.org/) was used to predict miRNA target genes. These genes were mapped to the Gene Ontology (GO) project (http://www.geneontology.org/) and the Kyoto Encyclopedia of Genes and Genomes (KEGG) pathways [17] behind it. Meanwhile, the significantly enriched GO terms were identified by* P* value ⩽ 0.001, and the* P* value cut-off was 0.01 for KEGG terms.

## 3. Results and Discussion

### 3.1. Overview of Small RNA Profiling

The sRNA profiles were generated by sequencing six tissues namely, heart, liver, spleen, lung, kidney, and muscle of FMD and their length distribution summarized in Table 1 and Figure S1. A total of 118 million reads were retrieved from the sequencing. After trimming, 103 million good quality reads (average 17 million reads per tissue; 87.87%) were obtained. The results indicated that majority of sRNAs in each library were between 20 and 24 nt in length, which is in line with the typical size range of small RNAs generated by Dicer. Moreover, the clean reads of sRNA (20–24 nt length) were most abundant in lung (93.3%), followed by liver (89.15%), kidney (87.6%), spleen (72.8%), heart (63%), and muscle (62.3%) shown in Figure S1.

Among the 103,261,451 good quality small RNAs (sRNAs), 6,622,520 were unique. To gain further insights in tissue distribution, we identified unique as well as all sRNAs in each tissue and summarized in Table 2. Total sRNAs for each tissue distributed as heart (16.70%), liver (15.06%), spleen (11.79%), lung (20.15%), kidney (19.89%), and muscle (16.40%). The proportion of unique sRNAs was accounted for heart (45.17%), spleen (26.44%), muscle (8.97%), liver (7.46%), kidney (6.23%), and lung (5.73%).

**Figure 1:**
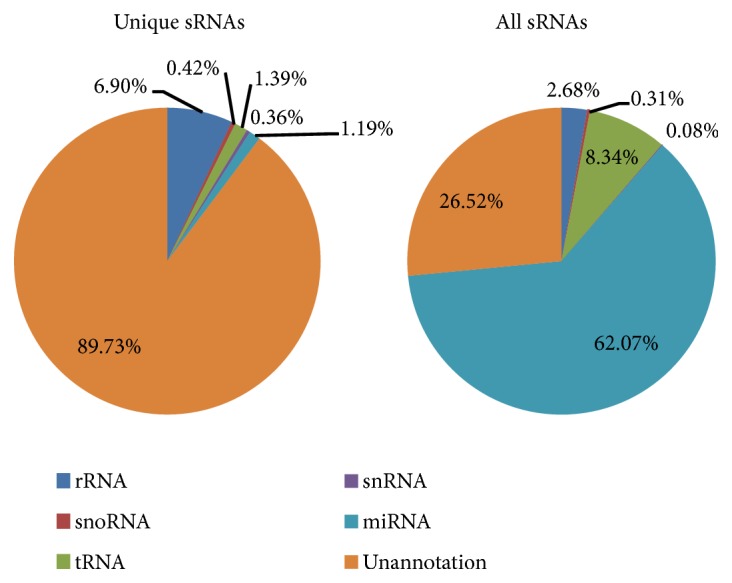
Summary of unique sRNAs and all sRNAs in six tissues of musk deer.

To provide more comprehensive information, we determined the distribution and frequency for each sRNAs types within unique as well as all sRNAs (see Figure 1). The result showed that, among unique sRNAs, the miRNA account for a small proportion (1.19%), and the most sRNA were found unannotated (89.73%). On the other hand, in all sRNAs, miRNA contributes major (62.07%) among all sRNAs followed by unannotated RNAs (26.52%).

**Figure 2:**
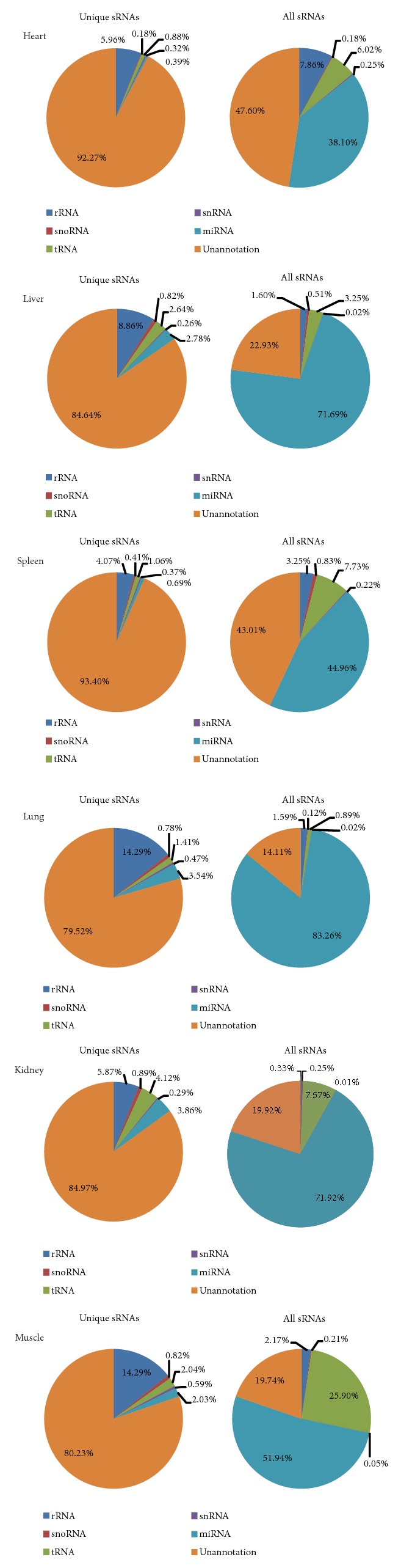
Pie chart of six tissues' unique and total sRNAs.

The detailed data illustrated that miRNAs are major composition of all sRNAs in each tissue samples. Small RNA-seq data contained significant representation of miRNAs in most of the tissues, with the maximum amount in the lung (83.26%), kidney (71.92%), and liver (71.69%) while miRNAs make up a tiny portion of unique sRNAs (less than 4%) shown in Table 3 and Figure 2. The tRNAs were maximum in small RNA-seq data of muscle (25.90%), followed by spleen (7.73%), kidney (7.57%), and heart (6.02%) and minimum in lung (0.89%). rRNA, snoRNA, and snRNAs were the minimal RNA species in all the tissues ranging between 0.33-7.86%, 0.12-0.83%, and 0.01-0.59%, respectively. The percentage of unannotated mapped reads was the highest in heart (47.60%) and the lowest in lung (14.11%). With a proportion being more than 79%, the unannotated RNA shares the major composition of unique sRNAs in all samples.

### 3.2. Comprehensive Analysis of miRNAs Expression Profiles

**Figure 3:**
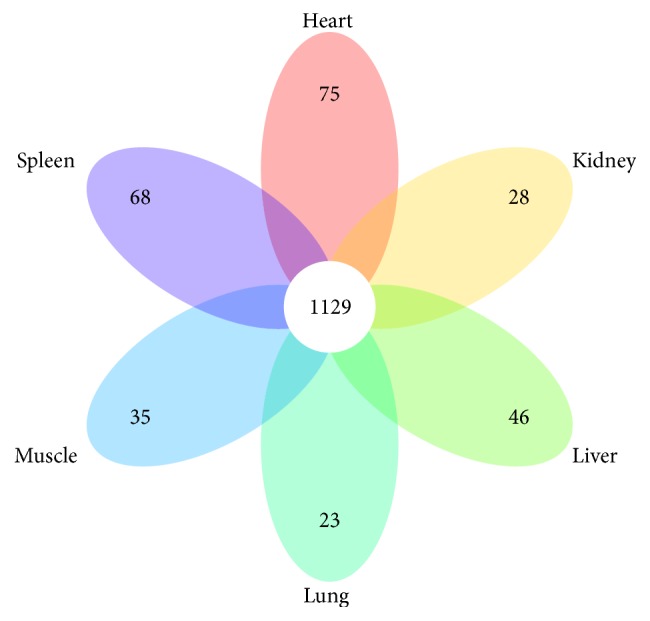
Comparison of miRNAs among six tissues.

In the present study, a total of 2,890 known miRNAs were identified in all six tissues. Among them, 1,129 (39.066%) known miRNAs were found to be coexpressed (see Figure 3). In the known miRNA expression profile, the reads numbers of the top 20 miRNAs accounted for each tissue as heart (51.028%), liver (53.988%), spleen (48.736%), lung (41.653%), kidney (45.090%), and muscle (44.028%). The expression profile indicated that a small portion of miRNA genes expresses most miRNAs.

The miRNA expression was normalized based on reads of exon model per million mapped reads (RPM) values. The expression of miRNAs, having RPM value more than 2000, was defined as highly coexpressed among examined tissue. Furthermore, 20 miRNAs were found to be highly coexpressed in all six tissues, and this suggested the crucial role of them for forest musk deer physiological processes (see Table 4).

We also sought to determine miRNAs having the highest expression in individual tissue examined in this study and displayed top five expressed miRNAs in each tissue (see Table 4). We found that five miRNAs belong to miRNA133 family (mir-133) highly expressed only in heart, which are efu-miR-133-3p, chi-miR-133a-3p, dme-miR-133-3p, gga-miR-133c-3p, and mmu-miR-133a-3p. Their average RPM was 68905.58 and efu-miR-133-3p had the highest RPM of 109983.53. We also found that mmu-miR-122-5p, mmu-miR-146a-5p, bta-miR-30a-5p, and mdo-miR-10b-5p had higher expression in liver, spleen, lung, and kidney, respectively. There were three miRNAs (hsa-miR-99a-5p, efu-miR-99a, and gga-miR-26a-5p) with high expression in muscle tissue. Furthermore, oha-miR-10c-5p found to be selectively coexpressed in spleen, kidney, and muscle with higher proportion. Besides highly expressed miRNAs, some were found to be expressed at very low level (RPM<10). This suggested the basal and extensive role of them for forest musk deer physiological processes.

### 3.3. Differential Expression of miRNAs

**Figure 4:**
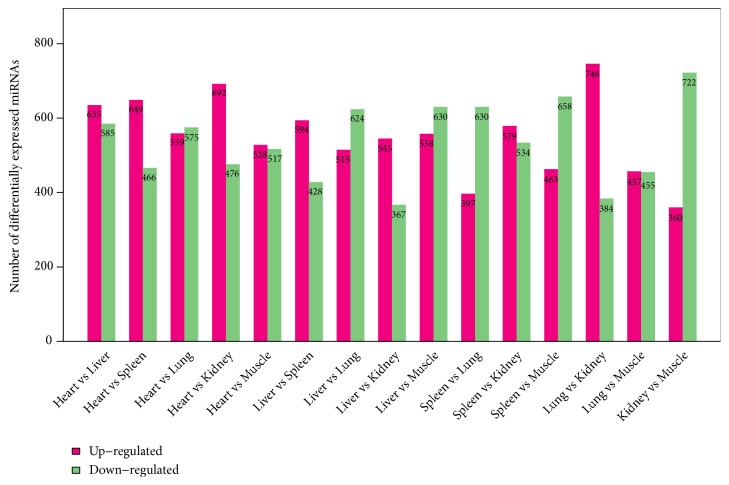
Level of differentially expressed miRNAs between different FMD tissues. In this figure, each pair of individuals compared is plotted on the x-axis, and the number of differently expressed miRNAs is plotted on the y-axis. In “1 versus 2” group, the red column represents miRNAs upregulated in the 1 compared to the 2 group, and the green column represents miRNAs downregulated in the 1 compared to the 2 group.

In order to identify differentially expressed miRNAs between tissues of musk deer, known miRNAs were compared in pairs to identify differentially expressed miRNA by multiple hypothesis testing. The results displayed that the number of differentially expressed miRNAs (*P*≤ 0.05) ranged from 912 (Lung versus Muscle and Liver versus Kidney) to 1,220 (Heart versus Liver), shown in Figure 4.

### 3.4. Gene Functional Annotation

In order to explore the function of target genes of these differentially expressed miRNAs, we mapped all of the target genes to terms in the GO and KEGG databases. Interestingly, in “Heart versus Liver” group, the GO enrichment analysis shows that the target genes were functionally enriched in organelle, cytoplasm, single−organism process, biological regulation, and others, although the functional enrichment in the other tissues were almost the same (see Figure S2).

KEGG pathway analysis of these target genes in all fifteen groups revealed that they were mainly regulating endocytosis, hedgehog signaling pathway, glycosaminoglycan biosynthesis-chondroitin sulfate/dermatan sulfate, and PI3K-Akt signaling pathway. The significant pathways (*P*<0.01) in each group are listed in Table S1.

Next-generation sequencing (NGS) technology is an advance method for analyzing transcriptome and has been extensively used to explore several kinds of miRNAs and their role in many biological processes [18]. Based on this technology, a number of transcriptome data have been revealed from organisms [19]. Transcriptome data can provide a convenience for further functional researches. Previous study has reported the transcriptome expression of Chinese giant salamander liver, spleen, and muscle using NGS technology [20], as a representative example in rare animals.

In this study, high-throughput sequencing of small RNA was extensively used to characterize specific miRNA and differential expressions of small RNAs from several tissues of forest musk deer. We identified a total of 103,261,451 (~87.87%) good quality small RNA reads; of them 6,622,520 were unique across all tissues. The small RNAs in each library have most clean reads between 20 and 24 nt in length. Moreover, 30.5% unannotated sRNA clean reads of 30-33 nt were identified specific to muscle tissue (see Figure S1). Moreover, tRNA and its derived small RNAs account a large proportion of the all small RNAs of muscle tissue. Previously, some studies on mature mouse sperm reported novel tRNA-derived small RNAs which were 30–34 nt in length [21] and sperm tRNA-derived small RNAs may be a paternal epigenetic factor, which have effects on the development of fertilized egg [22]. Nonetheless, the diversity of small noncoding RNAs of mammalian viscera provides an opportunity to identify a specific subset of RNAs from muscle tissue, certainly warrants further investigation.

Intriguingly, we found 20 microRNAs were coexpressed in all six tissues and this suggests that vital role of them for FMD biological processes. Most abundant cluster of microRNA in all six tissues was predicted as miR-26 family (see Table 4), which was recently proven to be crucial roles in numerous biological processes such as cell proliferation, apoptosis, tumorigenesis at different stages of nontumor diseases, growth and development of normal tissues, and other biological processes by regulating some complex signaling pathway [23, 24]. The downregulation of miR-26 was observed in many tumor types, such as bladder cancer and breast cancer, whereas ectopic expression of miR-26 inhibits proliferation and decreases in various tumor types [25–29]. There are also some studies that revealed that the expression of miR-26 was upregulated in tumors such as glioma [30].

Let-7miRNA family also has various functions; it has been reported to control cell growth as a “posttranscriptional gatekeeper” of certain genes [31] and therefore likely represents a potential biomarker. The downregulation of let-7 in breast cancer cell lines caused let-7 to lose its ability to restrain Ras mRNA, resulting in the activation of p-Ras and p-ErK, as reported in a study by Sun et al. [32]. A recent study in humans further reported that the association between the loss of let-7 expression and metastatic events is so strong that may indicates the potential prognostic role of let-7 in patient stratification and, hence, optimum selection for treatment strategies [33].

Furthermore, miR-24-3p was shown to play a vital role as an oncogenic miRNA in lung cancer [34]. Highly expressed miR-191 is a key regulator of naive, memory, and regulatory T cell homeostasis [35]. Expression of the miR-99 family of microRNAs had been shown to be related to radiation sensitivity [36], proliferation of c-Src-transformed cells through targeting mTOR, and prostate cancer cells that are inhibited by miR-99a and miR-99b [37, 38]. miR-100-5p is a tumor oncogenic and could be used as a diagnostic biomarker for renal cell carcinoma [39]. Moreover, 11 miRNAs were found to be high expression in specific tissue. In them, miR-133 family (efu-miR-133-3p, chi-miR-133a-3p, dme-miR-133-3p, gga-miR-133c-3p, and mmu-miR-133a-3p) showed specifically high expression in heart, which has been function in the cardiomyocytes proliferation and suppresses muscle gene expression in the heart, cardiac development, and apoptosis [40, 41]. A number of miRNAs have been discovered that play a key role in regulating liver development, regeneration, and metabolic functions. miR-122 is known as a biomarker of hepatic disorders such as chronic hepatitis, nonalcoholic fatty-liver disease, and drug-induced liver disease [42]. Some recent studies indicated that rat liver miR-122 expression may be upregulated by bisphenol A, while doses of crocin can downregulate miR-122 expression in rat with hepatic injuries inducing by ischemia-reperfusion or bisphenol A [43, 44].

Moreover, studies showed that miRNA-146 negatively regulates the production of proinflammatory cytokines via NF-*κ*B signaling in human gingival fibroblasts [45], miR-26a regulates tissue, and cell growth and differentiation by acting to posttranscriptionally repress Zeste homolog 2 [46].

## 4. Conclusions

In summary, the study reveals the first comprehensive transcriptome profile in six tissues (heart, liver, spleen, lung, kidney, and muscle) of Chinese forest musk deer. We have identified several differentially expressed microRNAs and they were mainly implicated in endocytosis, hedgehog signaling pathway, glycosaminoglycan biosynthesis-chondroitin sulfate/dermatan sulfate, and PI3K-Akt signaling pathway. The dataset of assembled FMD unigenes should serve as advance in the research of forest musk deer biology and further contribute to forest musk deer breeding and conservation. Nevertheless, the validation of the relationship between forest musk deer miRNAs and target mRNAs in the regulation of specific physiological processes needs further explored.

## Figures and Tables

**Table 1:** Overview of small RNA sequencing data.

Sample	Raw data Reads	Clean data Reads	Clean ratio
Heart	24433973	17240398	70.56%
Liver	16464176	15555722	94.48%
Spleen	16050145	12177403	75.87%
Lung	21856274	20807731	95.20%
Kidney	21372529	20543764	96.12%
Muscle	17834070	16936433	94.97%

**Table 2:** Percent of small RNA among different tissues (based on clean reads).

Sample	Total	Heart	Liver	Spleen	Lung	Kidney	Muscle
Unique	6622520	2991694	494214	1750874	379506	412477	593755
	100.00%	45.17%	7.46%	26.44%	5.73%	6.23%	8.97%

All	103261451	17240398	15555722	12177403	20807731	20543764	16936433
	100.00%	16.70%	15.06%	11.79%	20.15%	19.89%	16.40%

**Table 3:** Percent of sRNA among different category (based on clean reads).

Tissue	Type	All sRNAs	rRNA	snoRNA	tRNA	snRNA	miRNA	Unannotated RNA
Heart	Unique	2991694	178449	5254	26218	9526	11791	2760456
	All	17240398	1355167	30608	1038181	42428	6568238	8205775
Liver	Unique	494214	43773	4042	13027	1306	13752	418314
	All	15555722	249141	78627	506245	2680	11152053	3566977
Spleen	Unique	1750874	71245	7135	18557	6525	12135	1635277
	All	12177403	395168	100512	941798	26462	5475547	5237916
Lung	Unique	379506	54224	2979	5335	1774	13417	301778
	All	20807731	331761	24979	185274	3960	17325383	2936376
Kidney	Unique	412477	24211	3679	17015	1182	15917	350474
	All	20543764	68135	50854	1555520	2512	14774111	4092633
Muscle	Unique	593755	84825	4890	12111	3515	12056	476358
	All	16936433	366789	34779	4386652	8721	8796589	3342903

**Table 4:** Details of miRNAs co-expressed in six tissues and top five expressed miRNAs in each tissue (based on RPM values).

miRNAs id	Heart	Liver	Spleen	Lung	Kidney	Muscle
*Highly co-expressed in six tissues*						
gga-miR-26a-5p	11845.96	14163.91	9089.65	14605.46	13174.14	22955.88
bta-miR-26c	9197.39	8849.19	5385.60	20763.60	8728.05	22296.45
hsa-miR-26a-5p	9197.39	8849.19	5385.60	20763.60	8728.05	22296.45
efu-miR-26c	8479.38	7642.24	4901.26	19425.71	7497.93	20205.52
efu-miR-26a	8477.56	7639.91	4900.16	19425.54	7497.25	20205.30
hsa-miR-24-3p	10735.44	3935.81	5138.27	20911.63	10985.37	10530.11
mmu-let-7i-5p	6011.89	3941.15	9824.74	5308.81	8154.78	8471.12
prd-let-7-5p	4961.80	2406.46	3513.49	2609.04	3739.98	6063.69
bfl-let-7b	4631.88	2019.67	3145.49	2379.40	3354.95	5730.55
mmu-let-7g-5p	4892.67	4929.86	8377.29	12722.68	7957.06	8324.51
cel-let-7-5p	4631.88	2019.67	3145.49	2379.40	3354.95	5730.55
hsa-let-7f-5p	3696.81	2536.01	8086.10	6563.00	3985.78	7358.84
efu-let-7f	3328.52	2084.62	7213.50	5682.56	3428.19	6283.42
xtr-miR-191	4134.62	2374.33	16347.50	10255.20	2707.55	4556.46
mmu-miR-191-5p	11285.40	4264.84	18377.98	17034.85	3703.62	12268.43
ipu-miR-99b	5455.89	3204.21	6936.32	25644.56	6671.23	53352.97
hsa-miR-99a-5p	3460.83	3182.96	5974.04	12059.75	4399.42	26706.39
hsa-miR-100-5p	9219.68	3634.46	2363.19	3966.19	3807.58	2970.57
mmu-miR-143-3p	5691.59	63829.92	68761.15	12924.51	31205.52	14902.92
mmu-miR-199a-5p	2350.30	6545.12	3991.08	6293.55	2766.45	9807.79
*Top five expressed miRNAs in each tissue highlighted in italic font*						
efu-miR-133-3p	*109983.53 *	18.02	62.47	45.98	20.25	31.49
chi-miR-133a-3p	*104197.43 *	18.74	58.45	42.08	20.28	31.49
dme-miR-133-3p	*51506.75 *	12.72	29.64	18.03	11.66	15.68
gga-miR-133c-3p	*50521.48 *	8.82	31.39	14.18	13.78	14.66
mmu-miR-133a-3p	*28318.71 *	5.29	19.11	10.82	7.10	8.87
mmu-miR-143-3p	5691.59	*63829.92 *	*68761.15 *	12924.51	*31205.52 *	14902.92
mmu-miR-122-5p	18.99	*45503.54 *	2.26	10.63	69.56	0.58
oar-miR-21	467.29	*44968.48 *	27937.50	*37154.37 *	11245.31	14944.98
bta-miR-21-5p	452.37	*42697.11 *	26743.64	*38786.14 *	10894.32	14227.14
sha-miR-21	452.98	*42666.35 *	26733.78	*38777.36 *	10893.04	14231.92
mmu-miR-146a-5p	1021.28	12466.74	*49110.55 *	16194.33	1743.54	7839.63
oha-miR-10c-5p	120.31	118.08	*38780.43 *	364.30	*64778.22 *	*23049.50 *
mmu-miR-10b-5p	93.51	97.50	*30870.99 *	312.27	*54405.99 *	20502.78
gga-miR-10b-5p	83.67	94.15	*29214.92 *	304.63	*51356.45 *	18684.49
ipu-miR-99b	5455.89	3204.21	6936.31	*25644.56 *	6671.23	*53352.97 *
bta-miR-30a-5p	3376.20	18395.86	4168.85	*22726.64 *	23668.16	1826.19
mdo-miR-10b-5p	79.71	92.90	28299.03	298.69	*49524.19 *	17945.32
hsa-miR-99a-5p	3460.83	3182.96	5974.04	12059.75	4399.42	*26706.39 *
efu-miR-99a	2806.55	1589.80	3737.55	11207.57	3321.49	*24994.14 *
gga-miR-26a-5p	11845.96	14163.91	9089.65	14605.46	13174.14	*22955.88 *

## Data Availability

The transcriptome data are available at NCBI with the accession number PRJNA541415.

## Supplementary Materials

Supplementary Materials Table S1. Significant pathway* (P< 0.01)* annotation of the target genes of differentially expressed miRNAs. Figure S1. Length distribution of six tissues of small RNAs. Figure S2. Details of GO annotations.
