# Supplementary material for: microRNA and Other Small RNA Sequence Profiling across Six Tissues of Chinese Forest Musk Deer (Moschus berezovskii)
Source: Biomed Res Int. 2019 May 12;2019:4370704. doi: 10.1155/2019/4370704 (PMC6535825; doi:10.1155/2019/4370704)
Supplement: Supplementary Materials — Table S1. Significant pathway (P< 0.01) annotation of the target genes of differentially expressed miRNAs. Figure S1. Length distribution of six tissues of small RNAs. Figure S2. Details of GO annotations. [file 4370704.f1.docx]

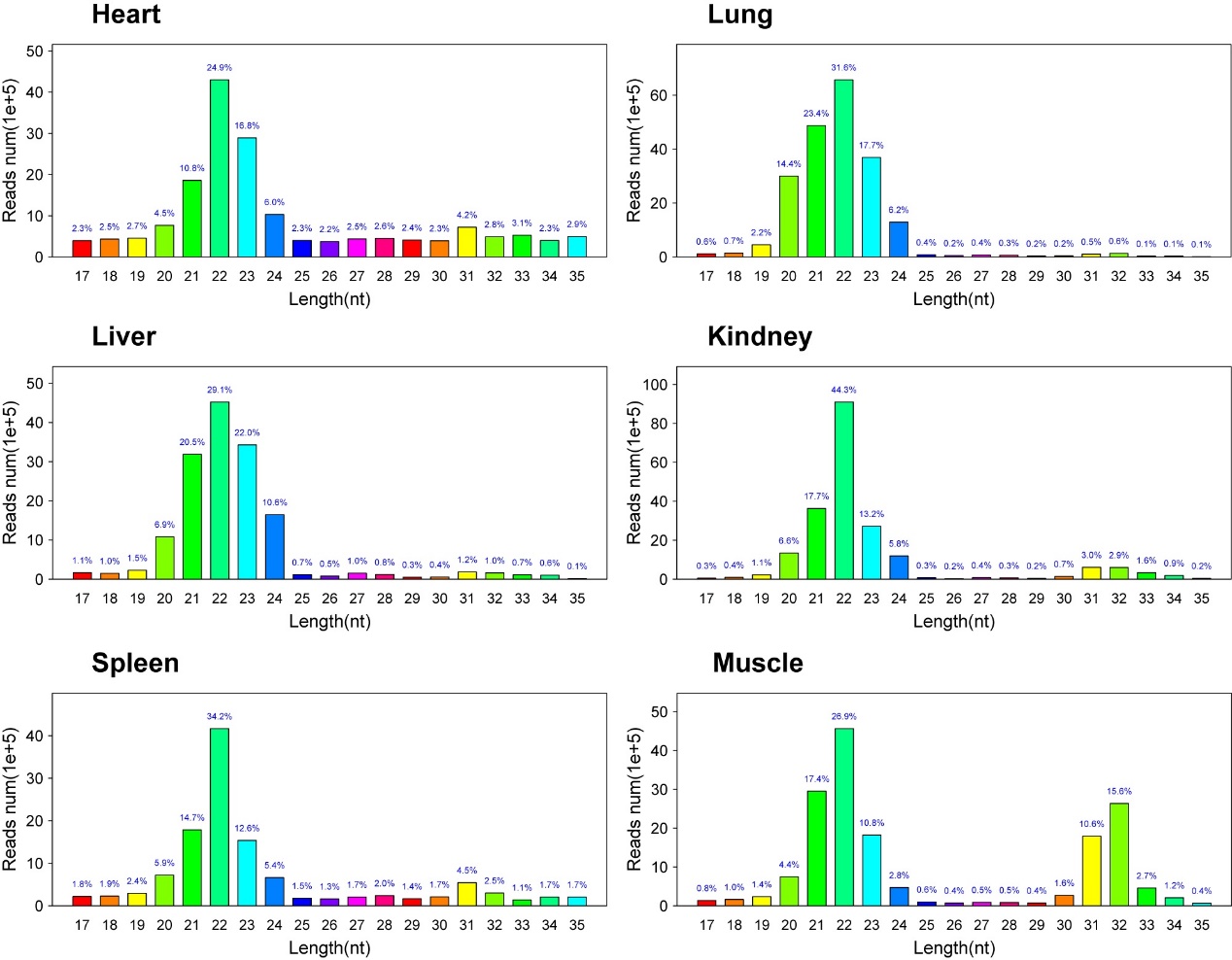


Figure S1. Length distribution of six tissues of small RNAs.


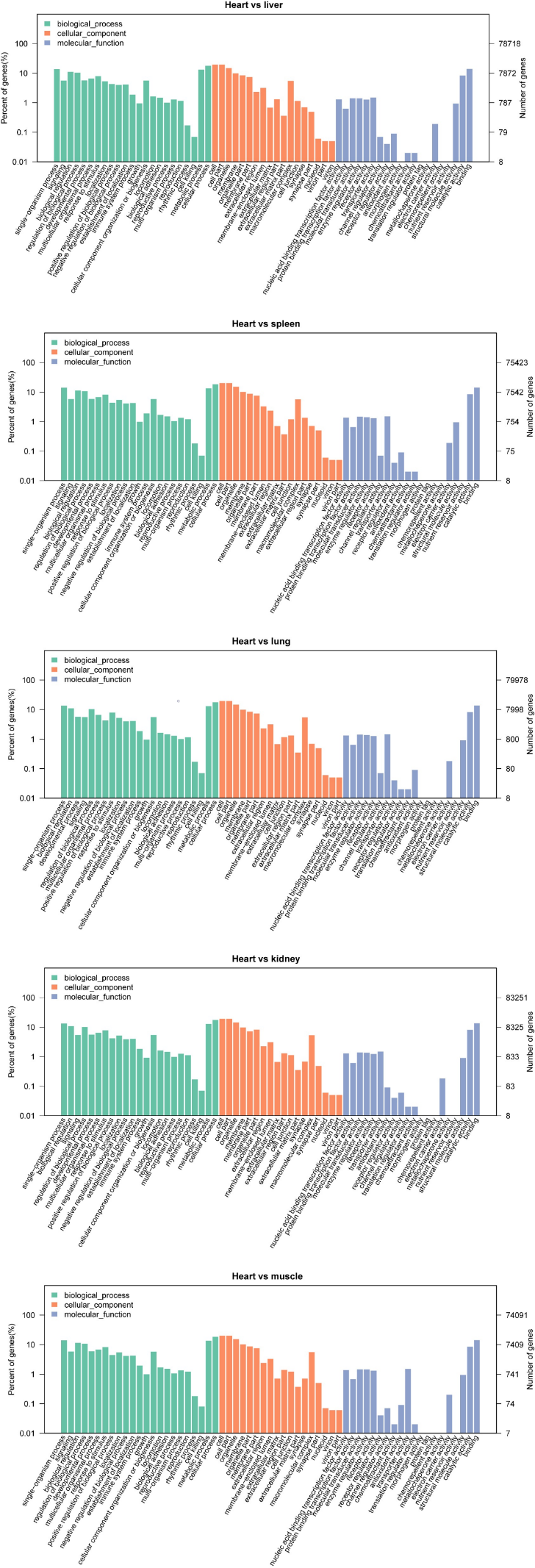

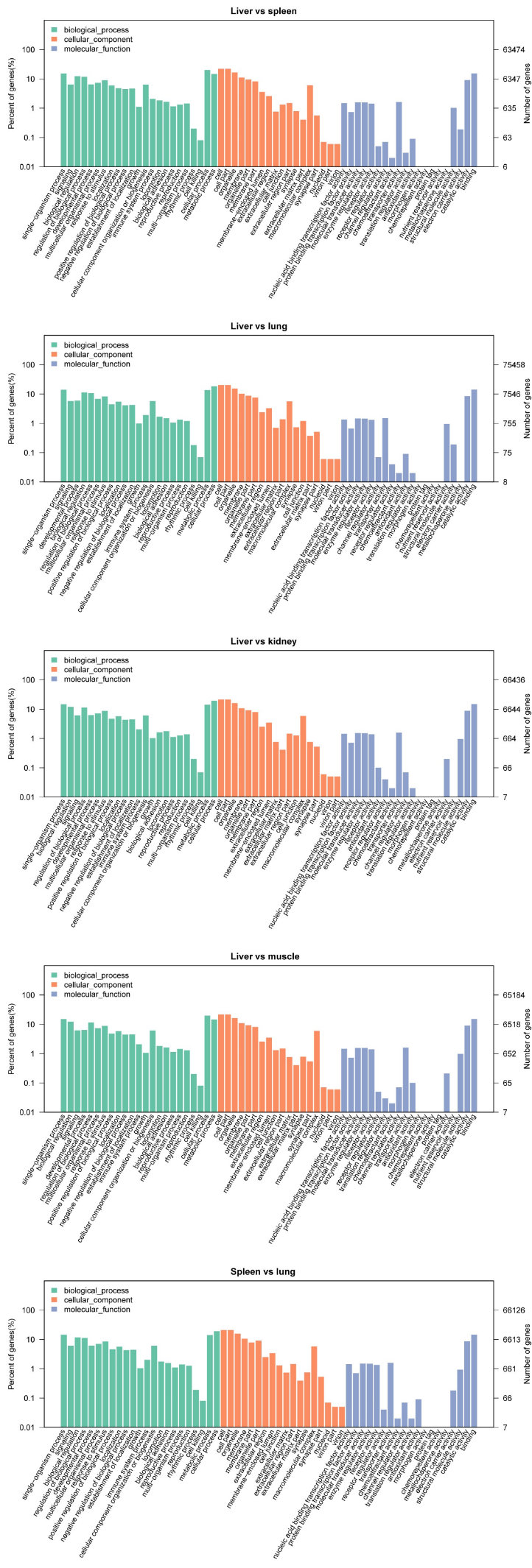

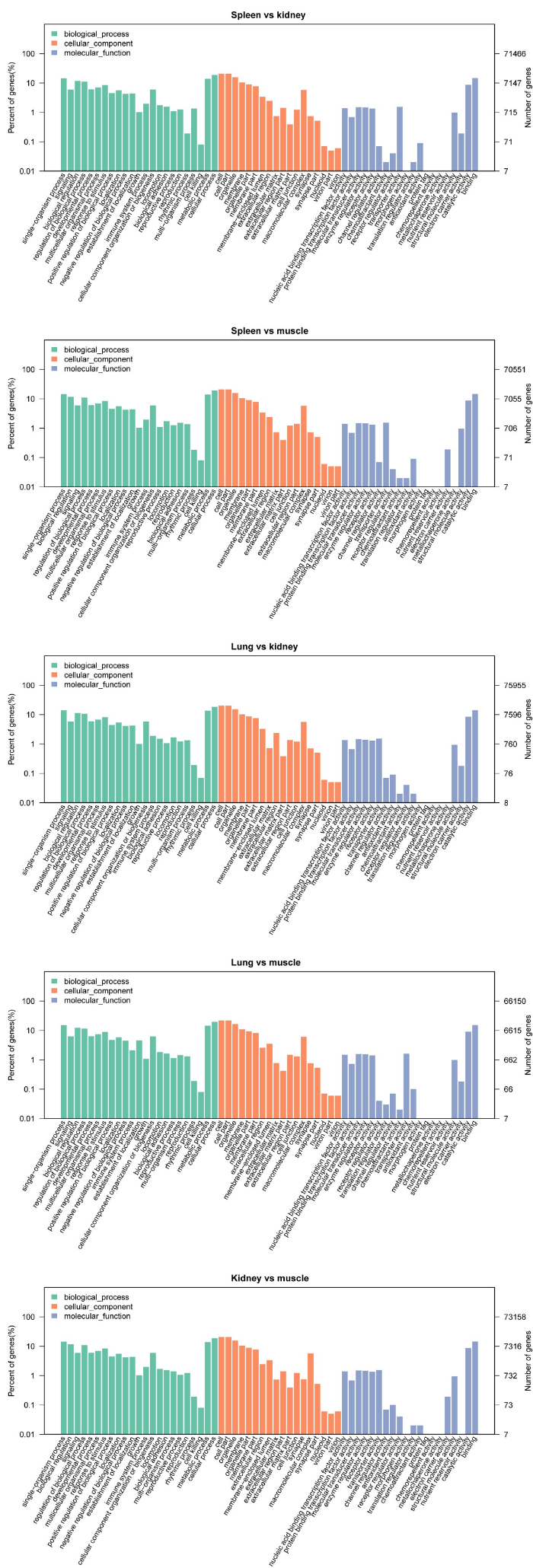


Figure S2. Details of GO annotations

Table S1. Significant pathway (*P*< 0.01) annotation of the target genes of differentially expressed miRNAs.

| Group | KO_ID | No. genes | P value | Term |
| --- | --- | --- | --- | --- |
| Heart  vs  Liver | ko04144 | 188 | 0.000227 | Endocytosis |
|  | ko04151 | 274 | 0.000373 | PI3K-Akt signaling pathway |
|  | ko04340 | 42 | 0.000655 | Hedgehog signaling pathway |
|  | ko04974 | 76 | 0.001542 | Protein digestion and absorption |
|  | ko05217 | 46 | 0.003517 | Basal cell carcinoma |
|  | ko04022 | 130 | 0.004777 | cGMP - PKG signaling pathway |
|  | ko00532 | 19 | 0.005626 | Glycosaminoglycan biosynthesis - chondroitin sulfate / dermatan sulfate |
|  | ko04930 | 43 | 0.006321 | Type II diabetes mellitus |
|  | ko04310 | 116 | 0.009514 | Wnt signaling pathway |
| Heart  vs  Spleen | ko04151 | 278 | 0.000005 | PI3K-Akt signaling pathway |
|  | ko04340 | 42 | 0.000376 | Hedgehog signaling pathway |
|  | ko04974 | 76 | 0.000715 | Protein digestion and absorption |
|  | ko04022 | 131 | 0.000897 | cGMP - PKG signaling pathway |
|  | ko04930 | 44 | 0.000969 | Type II diabetes mellitus |
|  | ko04920 | 61 | 0.002009 | Adipocytokine signaling pathway |
|  | ko04010 | 187 | 0.002034 | MAPK signaling pathway |
|  | ko04910 | 124 | 0.003991 | Insulin signaling pathway |
|  | ko04911 | 61 | 0.004648 | Insulin secretion |
|  | ko04710 | 31 | 0.005276 | Circadian rhythm |
| Heart  vs  Lung | ko04151 | 278 | 0.000099 | PI3K-Akt signaling pathway |
|  | ko04514 | 116 | 0.000843 | Cell adhesion molecules (CAMs) |
|  | ko04510 | 194 | 0.002543 | Focal adhesion |
|  | ko04340 | 41 | 0.004005 | Hedgehog signaling pathway |
|  | ko04010 | 189 | 0.004260 | MAPK signaling pathway |
|  | ko05202 | 134 | 0.004734 | Transcriptional misregulation in cancers |
|  | ko00532 | 19 | 0.006479 | Glycosaminoglycan biosynthesis - chondroitin sulfate / dermatan sulfate |
|  | ko04722 | 112 | 0.008484 | Neurotrophin signaling pathway |
|  | ko04710 | 31 | 0.009413 | Circadian rhythm |
| Heart  vs  Kidney | ko04151 | 276 | 0.001224 | PI3K-Akt signaling pathway |
|  | ko04340 | 42 | 0.001330 | Hedgehog signaling pathway |
|  | ko04710 | 32 | 0.002468 | Circadian rhythm |
|  | ko04010 | 190 | 0.006602 | MAPK signaling pathway |
|  | ko05217 | 46 | 0.006877 | Basal cell carcinoma |
|  | ko04144 | 185 | 0.007084 | Endocytosis |
|  | ko04514 | 114 | 0.007804 | Cell adhesion molecules (CAMs) |
|  | ko00532 | 19 | 0.008169 | Glycosaminoglycan biosynthesis - chondroitin sulfate / dermatan sulfate |
|  | ko04974 | 75 | 0.009516 | Protein digestion and absorption |
| Heart  vs  Muscle | ko04151 | 273 | 0.000026 | PI3K-Akt signaling pathway |
|  | ko04340 | 41 | 0.001309 | Hedgehog signaling pathway |
|  | ko04910 | 124 | 0.001982 | Insulin signaling pathway |
|  | ko04930 | 43 | 0.002678 | Type II diabetes mellitus |
|  | ko04974 | 74 | 0.002860 | Protein digestion and absorption |
|  | ko04920 | 60 | 0.003555 | Adipocytokine signaling pathway |
|  | ko04710 | 31 | 0.003926 | Circadian rhythm |
|  | ko04010 | 184 | 0.003983 | MAPK signaling pathway |
|  | ko05217 | 45 | 0.004821 | Basal cell carcinoma |
|  | ko04012 | 78 | 0.005649 | ErbB signaling pathway |
|  | ko05202 | 130 | 0.006347 | Transcriptional misregulation in cancers |
| Liver  vs  Spleen | ko04151 | 265 | 0.000006 | PI3K-Akt signaling pathway |
|  | ko04010 | 182 | 0.000216 | MAPK signaling pathway |
|  | ko04144 | 176 | 0.000519 | Endocytosis |
|  | ko05217 | 45 | 0.000903 | Basal cell carcinoma |
|  | ko04022 | 125 | 0.000984 | cGMP - PKG signaling pathway |
|  | ko00532 | 19 | 0.001300 | Glycosaminoglycan biosynthesis - chondroitin sulfate / dermatan sulfate |
|  | ko04910 | 120 | 0.001314 | Insulin signaling pathway |
|  | ko05200 | 260 | 0.003172 | Pathways in cancer |
|  | ko04920 | 58 | 0.003578 | Adipocytokine signaling pathway |
|  | ko04514 | 107 | 0.003729 | Cell adhesion molecules (CAMs) |
|  | ko05161 | 119 | 0.003931 | Hepatitis B |
|  | ko04340 | 39 | 0.003975 | Hedgehog signaling pathway |
|  | ko05215 | 71 | 0.004122 | Prostate cancer |
|  | ko05218 | 53 | 0.004819 | Melanoma |
|  | ko04710 | 30 | 0.004922 | Circadian rhythm |
|  | ko04974 | 70 | 0.008770 | Protein digestion and absorption |
| Liver  vs  Lung | ko04151 | 280 | 0.000005 | PI3K-Akt signaling pathway |
|  | ko04340 | 42 | 0.000551 | Hedgehog signaling pathway |
|  | ko04010 | 189 | 0.001596 | MAPK signaling pathway |
|  | ko04022 | 131 | 0.001837 | cGMP - PKG signaling pathway |
|  | ko05205 | 200 | 0.002658 | Proteoglycans in cancer |
|  | ko05217 | 46 | 0.002986 | Basal cell carcinoma |
|  | ko04370 | 59 | 0.004425 | VEGF signaling pathway |
|  | ko04066 | 93 | 0.005803 | HIF-1 signaling pathway |
|  | ko04710 | 31 | 0.006910 | Circadian rhythm |
|  | ko05215 | 74 | 0.007490 | Prostate cancer |
|  | ko00590 | 51 | 0.007799 | Arachidonic acid metabolism |
|  | ko05223 | 50 | 0.009299 | Non-small cell lung cancer |
| Liver  vs  Kidney | ko04151 | 263 | 0.000031 | PI3K-Akt signaling pathway |
|  | ko04360 | 113 | 0.000604 | Axon guidance |
|  | ko04010 | 180 | 0.000921 | MAPK signaling pathway |
|  | ko04310 | 113 | 0.001055 | Wnt signaling pathway |
|  | ko04510 | 183 | 0.001379 | Focal adhesion |
|  | ko00532 | 19 | 0.001392 | Glycosaminoglycan biosynthesis - chondroitin sulfate / dermatan sulfate |
|  | ko05205 | 190 | 0.001773 | Proteoglycans in cancer |
|  | ko04512 | 90 | 0.003152 | ECM-receptor interaction |
|  | ko04974 | 71 | 0.004761 | Protein digestion and absorption |
|  | ko04710 | 30 | 0.005359 | Circadian rhythm |
|  | ko05202 | 125 | 0.007317 | Transcriptional misregulation in cancers |
|  | ko04910 | 117 | 0.009491 | Insulin signaling pathway |
|  | ko04920 | 57 | 0.009597 | Adipocytokine signaling pathway |
| Liver  vs  Muscle | ko04151 | 262 | 0.000096 | PI3K-Akt signaling pathway |
|  | ko04340 | 41 | 0.000318 | Hedgehog signaling pathway |
|  | ko05217 | 45 | 0.001212 | Basal cell carcinoma |
|  | ko04710 | 31 | 0.001307 | Circadian rhythm |
|  | ko00532 | 19 | 0.001533 | Glycosaminoglycan biosynthesis - chondroitin sulfate / dermatan sulfate |
|  | ko05202 | 128 | 0.001732 | Transcriptional misregulation in cancers |
|  | ko04919 | 105 | 0.001765 | Thyroid hormone signaling pathway |
|  | ko04910 | 120 | 0.002186 | Insulin signaling pathway |
|  | ko04930 | 42 | 0.002503 | Type II diabetes mellitus |
|  | ko04722 | 107 | 0.003604 | Neurotrophin signaling pathway |
|  | ko04916 | 69 | 0.004510 | Melanogenesis |
|  | ko04022 | 123 | 0.005529 | cGMP - PKG signaling pathway |
|  | ko04144 | 172 | 0.007415 | Endocytosis |
|  | ko04310 | 110 | 0.009112 | Wnt signaling pathway |
|  | ko04010 | 176 | 0.009127 | MAPK signaling pathway |
|  | ko04012 | 75 | 0.009130 | ErbB signaling pathway |
| Spleen vs  Lung | ko04151 | 262 | 0.000021 | PI3K-Akt signaling pathway |
|  | ko04010 | 181 | 0.000272 | MAPK signaling pathway |
|  | ko04910 | 121 | 0.000500 | Insulin signaling pathway |
|  | ko05205 | 191 | 0.000557 | Proteoglycans in cancer |
|  | ko05217 | 45 | 0.000775 | Basal cell carcinoma |
|  | ko04340 | 40 | 0.000950 | Hedgehog signaling pathway |
|  | ko04066 | 90 | 0.001192 | HIF-1 signaling pathway |
|  | ko00532 | 19 | 0.001195 | Glycosaminoglycan biosynthesis - chondroitin sulfate / dermatan sulfate |
|  | ko04150 | 55 | 0.002714 | mTOR signaling pathway |
|  | ko04919 | 103 | 0.003412 | Thyroid hormone signaling pathway |
|  | ko04974 | 71 | 0.003441 | Protein digestion and absorption |
|  | ko05215 | 71 | 0.003441 | Prostate cancer |
|  | ko04370 | 56 | 0.004584 | VEGF signaling pathway |
|  | ko05200 | 258 | 0.004673 | Pathways in cancer |
|  | ko04510 | 179 | 0.005347 | Focal adhesion |
|  | ko04930 | 41 | 0.005373 | Type II diabetes mellitus |
|  | ko04916 | 68 | 0.005971 | Melanogenesis |
|  | ko04911 | 58 | 0.006059 | Insulin secretion |
|  | ko05221 | 51 | 0.006281 | Acute myeloid leukemia |
|  | ko04144 | 170 | 0.007875 | Endocytosis |
|  | ko04022 | 121 | 0.008187 | cGMP - PKG signaling pathway |
|  | ko04270 | 91 | 0.008445 | Vascular smooth muscle contraction |
|  | ko04514 | 105 | 0.009760 | Cell adhesion molecules (CAMs) |
| Spleen vs  Kidney | ko04151 | 274 | 0.000015 | PI3K-Akt signaling pathway |
|  | ko04010 | 191 | 0.000062 | MAPK signaling pathway |
|  | ko04974 | 75 | 0.001144 | Protein digestion and absorption |
|  | ko04910 | 124 | 0.002033 | Insulin signaling pathway |
|  | ko04310 | 116 | 0.002126 | Wnt signaling pathway |
|  | ko04510 | 189 | 0.002352 | Focal adhesion |
|  | ko04921 | 129 | 0.002883 | Oxytocin signaling pathway |
|  | ko04144 | 180 | 0.002909 | Endocytosis |
|  | ko04022 | 128 | 0.003291 | cGMP - PKG signaling pathway |
|  | ko00532 | 19 | 0.003416 | Glycosaminoglycan biosynthesis - chondroitin sulfate / dermatan sulfate |
|  | ko04710 | 31 | 0.003967 | Circadian rhythm |
|  | ko05218 | 55 | 0.004048 | Melanoma |
|  | ko04916 | 71 | 0.004829 | Melanogenesis |
|  | ko04015 | 170 | 0.005970 | Rap1 signaling pathway |
|  | ko05205 | 194 | 0.008778 | Proteoglycans in cancer |
|  | ko04930 | 42 | 0.008919 | Type II diabetes mellitus |
|  | ko04920 | 59 | 0.009103 | Adipocytokine signaling pathway |
|  | ko04722 | 109 | 0.009137 | Neurotrophin signaling pathway |
|  | ko04960 | 37 | 0.009750 | Aldosterone-regulated sodium reabsorption |
| Spleen vs  Muscle | ko04151 | 269 | 0.000036 | PI3K-Akt signaling pathway |
|  | ko04910 | 126 | 0.000144 | Insulin signaling pathway |
|  | ko04010 | 186 | 0.000350 | MAPK signaling pathway |
|  | ko04022 | 129 | 0.000560 | cGMP - PKG signaling pathway |
|  | ko04930 | 43 | 0.001554 | Type II diabetes mellitus |
|  | ko00532 | 19 | 0.002480 | Glycosaminoglycan biosynthesis - chondroitin sulfate / dermatan sulfate |
|  | ko05200 | 268 | 0.002668 | Pathways in cancer |
|  | ko05215 | 73 | 0.003293 | Prostate cancer |
|  | ko04919 | 106 | 0.003485 | Thyroid hormone signaling pathway |
|  | ko04066 | 91 | 0.003762 | HIF-1 signaling pathway |
|  | ko04911 | 60 | 0.004095 | Insulin secretion |
|  | ko05202 | 129 | 0.004317 | Transcriptional misregulation in cancers |
|  | ko04916 | 70 | 0.005522 | Melanogenesis |
|  | ko05218 | 54 | 0.005960 | Melanoma |
|  | ko05205 | 192 | 0.006438 | Proteoglycans in cancer |
|  | ko04370 | 57 | 0.007121 | VEGF signaling pathway |
|  | ko05217 | 44 | 0.008582 | Basal cell carcinoma |
| Lung  vs  Kidney | ko04151 | 273 | 0.000136 | PI3K-Akt signaling pathway |
|  | ko04911 | 62 | 0.001810 | Insulin secretion |
|  | ko04340 | 41 | 0.002041 | Hedgehog signaling pathway |
|  | ko04974 | 75 | 0.002135 | Protein digestion and absorption |
|  | ko00532 | 19 | 0.004375 | Glycosaminoglycan biosynthesis - chondroitin sulfate / dermatan sulfate |
|  | ko04710 | 31 | 0.005554 | Circadian rhythm |
|  | ko04022 | 128 | 0.007137 | cGMP - PKG signaling pathway |
|  | ko04144 | 180 | 0.007457 | Endocytosis |
|  | ko04725 | 83 | 0.008826 | Cholinergic synapse |
| Lung  vs  Muscle | ko04151 | 266 | 0.000030 | PI3K-Akt signaling pathway |
|  | ko04910 | 122 | 0.001025 | Insulin signaling pathway |
|  | ko04066 | 91 | 0.001704 | HIF-1 signaling pathway |
|  | ko04310 | 113 | 0.002423 | Wnt signaling pathway |
|  | ko05223 | 49 | 0.004278 | Non-small cell lung cancer |
|  | ko04510 | 182 | 0.006297 | Focal adhesion |
|  | ko04012 | 76 | 0.006513 | ErbB signaling pathway |
|  | ko04916 | 69 | 0.006522 | Melanogenesis |
|  | ko05205 | 189 | 0.007909 | Proteoglycans in cancer |
|  | ko04974 | 71 | 0.008391 | Protein digestion and absorption |
|  | ko05215 | 71 | 0.008391 | Prostate cancer |
|  | ko04014 | 164 | 0.008499 | Ras signaling pathway |
|  | ko04144 | 173 | 0.008649 | Endocytosis |
|  | ko05218 | 53 | 0.008732 | Melanoma |
|  | ko05214 | 56 | 0.009812 | Glioma |
| Kidney vs  Muscle | ko04151 | 269 | 0.000426 | PI3K-Akt signaling pathway |
|  | ko04022 | 131 | 0.000564 | cGMP - PKG signaling pathway |
|  | ko04921 | 131 | 0.001011 | Oxytocin signaling pathway |
|  | ko04144 | 181 | 0.002403 | Endocytosis |
|  | ko04725 | 84 | 0.002722 | Cholinergic synapse |
|  | ko00532 | 19 | 0.003716 | Glycosaminoglycan biosynthesis - chondroitin sulfate / dermatan sulfate |
|  | ko04710 | 31 | 0.004449 | Circadian rhythm |
|  | ko04010 | 184 | 0.005658 | MAPK signaling pathway |
|  | ko04916 | 71 | 0.005812 | Melanogenesis |
|  | ko04340 | 40 | 0.005916 | Hedgehog signaling pathway |
|  | ko04974 | 73 | 0.008087 | Protein digestion and absorption |
|  | ko04310 | 114 | 0.009729 | Wnt signaling pathway |
|  | ko04270 | 95 | 0.009999 | Vascular smooth muscle contraction |
